# Advantage of *HSP110* (T17) marker inclusion for microsatellite instability (MSI) detection in colorectal cancer patients

**DOI:** 10.18632/oncotarget.25611

**Published:** 2018-06-19

**Authors:** Gustavo Noriz Berardinelli, Cristovam Scapulatempo-Neto, Ronílson Durães, Marco Antônio de Oliveira, Denise Guimarães, Rui Manuel Reis

**Affiliations:** ^1^ Molecular Oncology Research Center, Barretos Cancer Hospital, Barretos, São Paulo, Brazil; ^2^ Department of Oncology, Barretos Cancer Hospital, Jales, São Paulo, Brazil; ^3^ Nucleus of Epidemiology and Biostatistics, Barretos Cancer Hospital, Barretos, São Paulo, Brazil; ^4^ Department of Endoscopy, Barretos Cancer Hospital, Barretos, São Paulo, Brazil; ^5^ Life and Health sciences Research Institute, University of Minho, Gualtar Campus, Braga, Portugal; ^6^ ICVS/3B’s-PT Government Associate Laboratory, Gualtar Campus, Braga, Portugal

**Keywords:** colorectal cancer, microsatellite instability, molecular diagnostic, immunohistochemistry, *HSP110* (T17)

## Abstract

Colorectal cancer (CRC) is a leading cause of cancer death worldwide. Microsatellite instability (MSI) is a genetic pathway leading to CRC, associated with particular clinicopathological features, and recently a major biomarker of immunotherapy response. There is little information the frequency MSI among Brazilian CRC patients, and it is still debatable the ideal methodology for MSI screening in countries with limited resources. We proposed to evaluate MSI by molecular and immunohistochemistry (IHC) methods, to compare both methodologies and also to assess the inclusion of a novel microsatellite marker, *HSP110* (T17). The molecular MSI evaluation was performed using a PCR-multiplex panel in a total of 1013 CRC patients. Mismatch repair (MMR) proteins (MLH1, MSH2, MSH6 and PMS2) expression were evaluated by IHC. *HSP110* (T17) marker was analyzed by fragment analysis. Molecularly, 89.5% of cases were MSI-negative and 10.5% were MSI-positive. The IHC showed that 88.9% of cases exhibited MMR-proficient status, 10.2% were MMR-deficient and 0.9% was inconclusive. Genotyping of the *HSP110* (T17) in 106 MSI-positive and 215 MSI-negative cases showed its alteration only among the MSI-positive cases. We observed agreement (0.956, Kappa Test) between both molecular and IHC methodologies, with only eight discordant results, and in this subset of cases the *HSP110* (T17) corroborate the molecular findings. This study suggests the use of molecular assays over IHC for MSI analysis and proposes the inclusion *HSP110* (T17) marker as a complementary analysis in discordant cases.

## INTRODUCTION

Colorectal cancer (CRC) is a common diagnosis in high-income countries and its incidence is rising in middle-income countries, including Brazil [1–3]. In US, it is the second largest cause of cancer-related deaths when both sexes are combined [2]. Five-year survival rate is about 50%, with no differences between high-income countries (49% average) and low- and middle-income countries (44% average) [1]. In Brazil, CRC is the third most common cause of cancer for men and the second for women with approximately 30,000 new cases diagnosed per year for both genders [3]. The incidence rates are increasing due to population aging, increasing smoking rates, changes in diet style and the absence of wide spread screening programs [4].

Initiation and progression of CRC involve different molecular mechanisms responsible for distinct clinicopathological features and behavior of tumors [5]. A large body of evidence suggests that tumor location (left versus right) drives carcinogenesis [5, 6], and genomics abnormalities occur in a non-random pattern in the evolution of adenoma-carcinoma then metastasis [7]. Chromosomal instability (CIN), microsatellite instability (MSI) and CpG island methylation phenotype (CIMP) are genetical pathways involved in the development of CRCs affecting oncogenes, tumor suppressor genes and DNA repair mechanisms [7, 8]. More recently, CRC have been subtyped molecularly in four consensus molecular subtypes (CMSs) based on gene expression with distinguishing features: CMS1 (microsatellite instability immune, 14%), hypermutated, microsatellite unstable and strong immune activation; CMS2 (canonical, 37%), epithelial, marked WNT and MYC signaling activation; CMS3 (metabolic, 13%), epithelial and evident metabolic dysregulation; and CMS4 (mesenchymal, 23%), prominent transforming growth factor–beta activation, stromal invasion and angiogenesis [9].

MSI occurs when Mismatch Repair (MMR) proteins (MLH1, MLH3, MSH2, MSH3, MSH6, PMS1, and PMS2) are absent due to mutations or promoter hypermethylation in hereditary and sporadic forms, respectively [10, 11]. These DNA repair proteins are important to repair base-base mismatches occurring during DNA replication; thus their loss leads to an accumulation of DNA replication errors, particularly in areas of the genome with short repetitive nucleotide sequences, known as microsatellites [12]. Genes containing microsatellite regions are known as MSI-target genes and are involved in several functions related with carcinogenesis, such as DNA repair, DNA damage sensor, apoptosis, signalization, proliferation etc [11, 13]. As the alterations occur in a random matter, the findings suggest a different progression of each MSI-positive tumor, a model in which the MSI mutator phenotype develops in gradual steps by successive alterations of the different MSI-target genes [11, 14–16].

Hereditary Nonpolyposis Colorectal Cancer (HNPCC) or Lynch Syndrome is the most frequent hereditary colorectal cancer syndromes and is the result of the presence of germline mutations in *MMR* genes, mainly *MLH1* and *MSH2* with somatic inactivation of the remaining wild-type allele [10, 17]. On the other hand, in sporadic CRC, MSI phenotype is less frequent (12-15%) and is predominantly due to loss of *MLH1* function, caused by transcriptional silencing of the gene brought about by abnormal methylation of *MLH1* promoter region [18].

Clinically, MSI positive CRCs are associated with several features, such as proximal location, poorly differentiated histology, intense lymphocytic infiltration, favorable prognosis compared to MSI negative CRCs, and lower risk of metastasis [6, 19]. There are also several evidences suggesting that MSI positive patients are less responsive to 5-Fluorouracil-based chemotherapy (5-FU), and more responsive to irinotecan based regimens [19–22]. Recently, MSI phenotype was reported as a predictive biomarker of response to immunotherapy treatments, more specifically anti-PD-1 therapy [23, 24]. Therefore, MSI is a useful molecular marker not only for diagnostic and prognostic purposes, but also for therapy prediction response in CRC patients, being currently, a crucial biomarker for CRC management [24, 25].

Nowadays, immunohistochemistry (IHC) and molecular methods are the most common assays for MSI assessment in CRCs. IHC, will detect the loss expression of MMR proteins in tumor tissue compared to adjacent normal, thus resulting in presence or absence of MMR proteins expression (MMR-proficiency versus -deficiency) [22].

Molecularly, MSI status can be assessed by PCR analysis of informative microsatellite markers, either mononucleotide and dinucleotide repeats, which are further analyzed by capillary sequencing [26, 27]. An initial panel of genetic markers, known as Bethesda panel, was established at a National Cancer Institute’s (NCI) workshop, and includes two mononucleotide (BAT25 and BAT26) and three dinucleotide (D5S346, D2S123, and D17S250) repeats; this assay requires including matched-normal DNA as a reference [26]. Later, a subsequent NCI’s workshop re-evaluated the Bethesda panel and recommended the substitution of dinucleotide repeats by quasi-monomorphic mononucleotide repeat markers (BAT25, BAT26, NR21, NR24, and NR27), which allow performing the assay only in tumor DNA, thus avoiding the use of paired normal DNA [28]. This method classified tumors as high microsatellite instability (MSI-H) when at least two of five markers are instable, low microsatellite instability (MSI-L) when only one marker is instable and microsatellite stability (MSS) [27, 29].

Recently, we implemented an optimized molecular assay for MSI evaluation for the Brazilian population [27]. We established a quasi-monomorphic variation range (QMVR) for each marker in a Brazilian healthy population allowing the use of MSI markers without matching normal DNA, which was independent of the ethnicity, even in the highly admixed population of Brazil [27].

Recently it has been reported a high frequency of mutation of the *HSP110* microsatellite T17 (mononucleotide repeat retained in intron 8) in MSI-positive CRC cases [14]. Changes in this region have been shown to be important because of their association with biological effects leading to an increased synthesis of a variant *HSP110* isoform due to exon 9 skipping (HSP110DE9) [14, 15]. Chaperone proteins including HSP110 are expressed in specific set of stress conditions promoting the survival of malignant cells in colon cancers [30]. Deletions in *HSP110* (T17) were shown to increase the sensitivity of CRC cells to 5-Fluorouracil and oxaliplatin [15]. Therefore, analysis of *HSP110* (T17) was become an important tool for the identification of MSI-positive CRC cases and the appropriate clinical management of patients.

There is little information on MSI in Brazilian CRC patients and there is still some controversies concerning the ideal approach for MSI screening. So, in the present study we proposed to evaluate MSI by molecular and IHC methods in a large set of Brazilian CRC patients, to compare both methodologies, and to improve the analysis of discordant cases.

## RESULTS

### Determination of MSI phenotype by MMR immunohistochemistry

**Figure 1:**
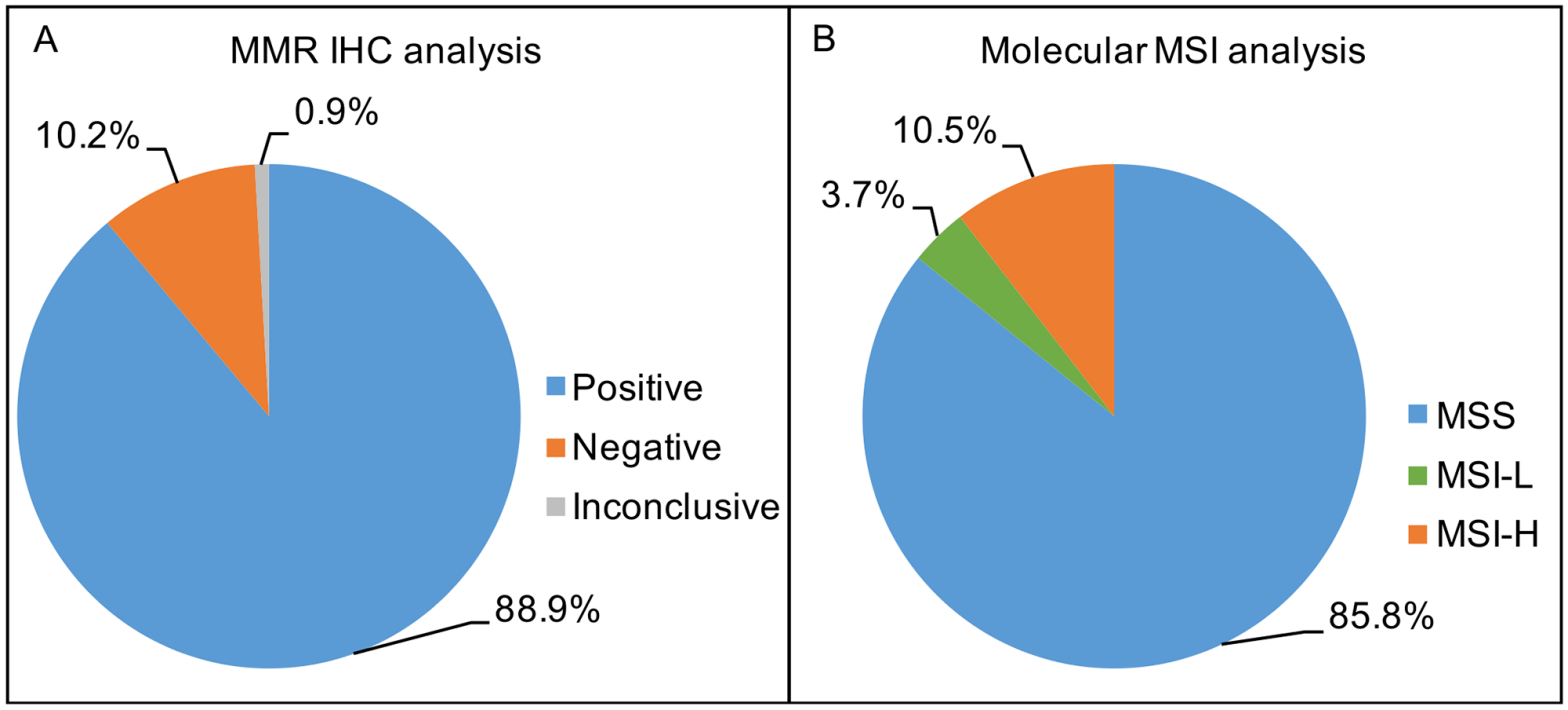
**(A)** Percentage of MMR IHC status for 996 Brazilian CRC patients; Positive IHC status: 88.9% (886/996), Negative IHC status: 10.2% (102/996) and Inconclusive IHC status 0.9% (8/996). **(B)** Percentage of molecular MSI status for 1013 Brazilian CRC patients; MSS (microsatellite stability): 85.8% (870/1013), MSI-L (low-microsatellite instability): 3.7% (37/1013) and MSI-H (high-microsatellite instability): 10.5% (106/1013).

Expression analysis of MMR proteins (MLH1, MSH6, MSH2 and PMS2) was performed in 996/1013 CRC cases by immunohistochemistry (IHC). In a total of 17 cases there was not enough biological material for the analysis. Among those cases, 88.9% (886/996) presented expression of the four MMR proteins, thus being considered positive, 10.2% (102/996) exhibited loss of expression of at least one of the four MMR proteins and thus considered negative, and 0.9% (8/996) were considered inconclusive since the internal positive controls were negative (Figure 1A). Among the negative IHC cases, 52.0% (53/102) presented loss of MLH1/PMS2 and 20.6% (21/102) loss of MSH2/MHS6. We also observed: 1.9%, 2.9%, 7.9% and 6.9% of cases presenting isolated loss of MLH1, MSH2, MSH6 and PMS2, respectively. Other combinations of loss protein expression were observed in 7.8% (8/102) of cases and none of cases showed loss of expression of the all four proteins.

### Determination of MSI phenotype by molecular analysis

**Figure 2:**
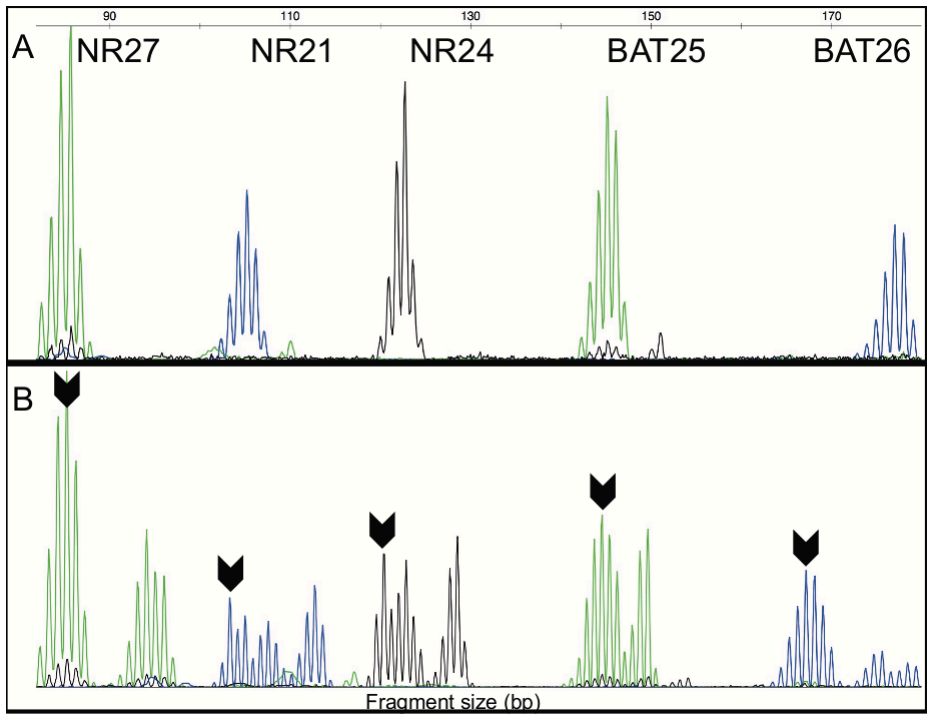
**(A)** Fragment analysis of peaks for molecular MSI analysis in one MSS CRC sample with five markers (NR27, NR21, NR24, BAT25 and BAT26) within of the quasi-monomorphic variation range (QMVR). **(B)** Fragment analysis of peaks for molecular MSI analysis in one MSI-H CRC sample with five markers (NR27, NR21, NR24, BAT25 and BAT26) outside of the QMVR. Arrow indicates the allele outside of the QMVR. bp – base pair.

All 1,013 CRC were analyzed for MSI by molecular analysis. We observed that 85.8% (870/1013) of cases were MSS, 3.7% (37/1013) were MSI-L and 10.5% (106/1013) were MSI-H (Figure 1B and Figure 2). We further grouped together the MSS and MSI-L cases, named: MSI-negative (MSS+MSI-L), while the MSI-H cases were named: MSI-positive [11, 16, 31].

### Validation, sensitivity, specificity and accuracy of molecular MSI analysis

Using IHC as gold standard to determine the microsatellite instability, the agreement analysis between the methodologies was performed by the Kappa Test (Table 1). Considering that positive IHC status should represent MSI-negative status and that negative IHC status corresponding to MSI-positive status, we observed that the Measure of Agreement between the techniques was 0.956 (p <0.001) (Table 1).

**Table 1:** Measure of Agreement (Kappa Test) between immunohistochemistry (IHC) and molecular MSI analyses

IHC status (n)	Molecular MSI status (n)	Measure of Agreement
MSI- (886)	MSI- (882)	0.956 (p<0,001)
	MSI+ (4)	
MSI+ (102)	MSI-(4)	
	MSI+ (98)	

+: positive, -: negative, n: number of cases

Hence, the sensitivity, specificity and accuracy of the molecular MSI analysis was: 99.5%, 96.1% and 99.2%, respectively (Table 2).

**Table 2:** Estimation of sensitivity, specificity and accuracy of molecular MSI analysis

	IHC-negative (n)	IHC-positive (n)	Total
**MSI-positive (MSI-H)**	98	4	102
**MSI-negative (MSS+MSI-L)**	4	882	886
**Total**	102	886	988

MSS: microsatellite stability, MSI-L: low-microsatellite instability, MSI-H: high-microsatellite instability, n: number of cases.

### Determination of *HSP110* (T17) QMVR and tumor genotyping

**Figure 3:**
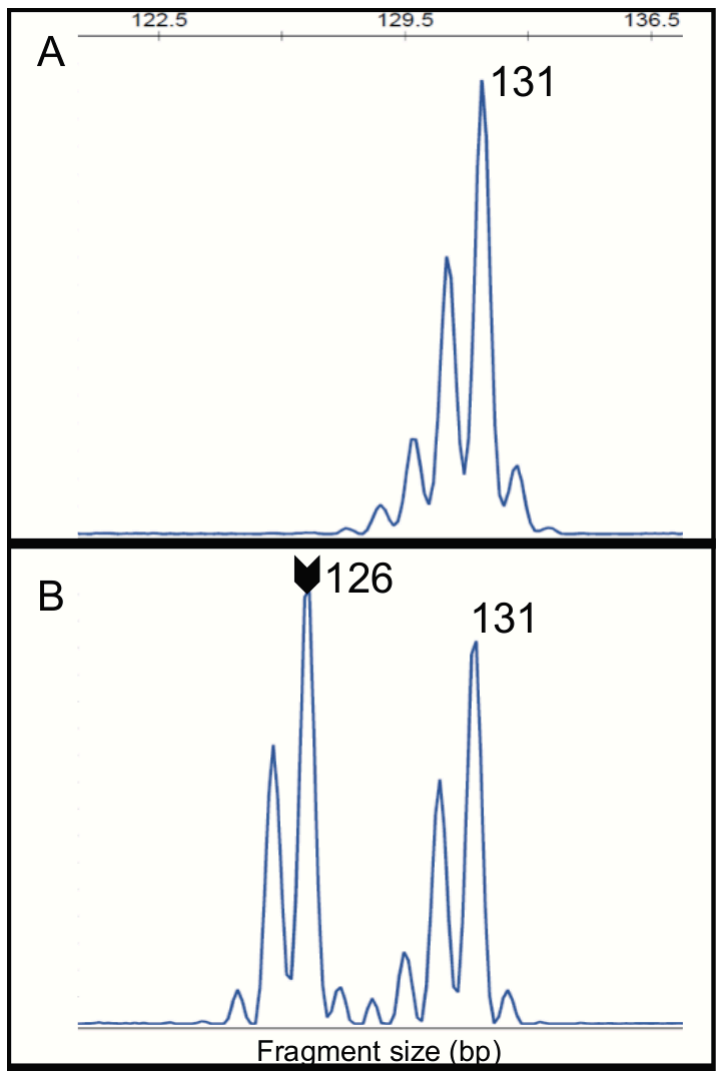
**(A)** The allele 131 of *HSP110* (T17) marker was the most observed in 54.7% (234/428) of analyzed alleles from healthy individuals blood DNA. **(B)** Two alleles (131 and 126) of *HSP110* (T17) marker were observed in MSI-H CRC tumor DNA showing alteration in this marker. Arrow indicates the allele outside of the QMVR. bp – base pair.

A recent report suggests *HSP110* (T17) has value as a complementary marker in MSI determination [32]. In order to evaluate the role *HSP110* (T17) in our cohort, we initially determined its quasi-monomorphic variation range (QMVR). For that, a total of 214 DNA from healthy subjects were analyzed to determine the normal range. All the 214 samples were successfully amplified, generating 428 alleles. The allele size was monomorphic with only two observed alleles: 131 and 132 base pairs. The 131 bp allele was observed in 234/428 (54.7%) alleles (Figure 3A) and the 132 base pair was seen in 194/428 (45.3%) alleles. Therefore, we determined the *HSP110* (T17) QMVR as 130-133 bp, since ±1 bp variations can occur from different reagents and equipment’s used in the analyses [28, 33].

Following the QMVR determination, we further genotyped the *HSP110* (T17) in the tumor tissue from CRC patients, namely in MSI-positive cases (MSI-H=106) and MSI-negative cases (subsets of MSS=180 and of MSI-L=35). In MSI-negative cases, all of them exhibited the *HSP110* (T17) within QMVR above determined. In MSI-positive cases, 7.6% (8/106) were inconclusive, due to very poor DNA quality. Among the conclusive cases we observed 5.1% (5/98) of cases without alterations, and 94.9% (93/98) with altered *HSP110* (T17), exhibiting alleles with losses of up to 6 bp in relation to the QMVR (Figure 3B) and aberrant peak ratios.

### Discordant IHC and molecular MSI results

Despite the high agreement between IHC and molecular methodologies, we observed discordant results in eight cases (Table 3, Supplementary Table 1). In these cases, we assessed the *HSP110* (T17) marker, which showed to be alterated in the four MSI-H/positive-IHC cases (Table 3, Supplementary Table 1). The two MSS/negative-IHC and two MSI-L/negative-IHC cases also showed presence of *HSP110* (T17) alteration (Table 3, Supplementary Table 1).

**Table 3:** Summary of cases with discordant data between immunohistochemistry and molecular MSI analysis

Sample ID	IHC status	MLH1	MSH2	MSH6	PMS2	Molecular MSI status	*HSP110* (T17) status
11.0011	negative	+	+	+	**-**	MSS	altered^a^
12.0767	negative	-	+	+	-	MSS	altered^a^
11.0226	negative	+	+	-	+	MSI-L	altered^a^
13.0395	negative	+	+	+	-	MSI-L	altered^a^
11.0404	positive	+	+	+	+	MSI-H	altered
11.0827	positive	+	+	+	+	MSI-H	altered
13.0578	positive	+	+	+	+	MSI-H	altered
13.1023	positive	+	+	+	+	MSI-H	altered

*HSP110* (T17) status obtained by comparison between normal and tumor tissue DNA from the samples and also by R method.

+: positive protein expression; -: negative protein expression; MSS: microsatellite stability; MSI-L: low-microsatellite instability; MSI-H: high-microsatellite instability; ^a^: analyzed by R method.

## DISCUSSION

In colorectal cancer (CRC), the presence of MSI has been used as a biomarker for several purposes: i) hereditary screening; ii) prognostic marker, where stage II/III tumors presenting MSI have a better prognosis than those that do not present it; iii) 5-FU resistance and irinotecan sensitivity [34]; and more recently iv) immunotherapy response [23, 24].

Therefore, the identification of MSI plays a major role in cancer management and different analytical methodologies are currently used, being immunohistochemistry (IHC) and molecular testing (microsatellite markers) the most widespread. Both techniques are suggested in the American Society of Clinical Oncology (ASCO) and National Comprehensive Cancer Network (NCCN) guidelines for evaluation of MSI [35, 36]. The methodology of choice can be associated a several factor, such as expertise of the laboratory, availability of technology and overall costs. In the present study we intended to compare different methodologies in a Brazilian routine setting. We analyzed 1,013 CRC cases and used a pentaplex PCR assay previously validated by our group in the Brazilian population [27]. We observed 85.8% of MSS cases, 3.7% of MSI-L cases and 10.5% of MSI-H cases. By IHC analysis, we observed that 10.2% of the cases exhibited MSI, with a Measure of Agreement (Kappa test) of 0.956 between both IHC and molecular methods. Besides the high level of agreement, the sensitivity, specificity and accuracy of the molecular test were 99.5%, 96.1% and 99.2%, respectively, when the IHC was used as the gold standard. Our results are in accordance with previous series. Patil *et al* [37] used a panel of five markers similar to those used in our study and the agreement between the techniques was 100%. Cicek *et al* [38] used a panel that included mononucleotide and dinucleotide markers and the cases identified by MSI and IHC were highly concordant when using the cutoff of 30% or greater for instable molecular markers. Zhang [39] compared both methods and concluded that molecular MSI is as sensitive and specific as IHC, given its excellent reproducibility and its potential capability to indicate mutations not be detected by IHC. Molecular MSI has been used and will continue to prevail as the primary screening tool for identifying HNPCC patients. To some extent, molecular MSI and IHC are complementary to each other in identifying HNPCC [39]. Lindor *et al* [40] evaluated both methods and the predictive value of abnormal IHC was 100% for an MSI-H phenotype and that testing strategies must take into account acceptability of missing some cases of MSI-H tumors if only IHC is performed. At variance, Lin *et al* [41] obtained 23 (32.9%) discordant cases for the same comparison between methodologies. The panel of molecular markers used by Lin *et al* [41] may justify this high percentage of discordance, where, dinucleotide markers are known to be less sensitive compared to mononucleotide markers [42], which were used in our study. In addition, it was been reported that the molecular analysis is less prone to change than IHC due to neoadjuvant and radiation therapy in CRC, therefore, the ideal methodology, to assess MSI status after neoadjuvant therapy [43].

Despite the high agreement among methodologies, we observed disagreement in eight cases. Recently, *HSP110* (T17) marker has been proposed as a complementary marker in MSI assessment [32]. Therefore, we initially determined the QMVR of *HSP110* (T17) in a Brazilian healthy population. This analysis showed the monomorphic nature of the marker. We further assess this marker in MSI-positive cases and observed 94.9% with altered *HSP110* (T17). This value was as high as the values obtained by Dorard *et al* [14], Collura *et al* [15], Buhard *et al* [32] and Markovic *et al* [44] of *HSP110* (T17) changes in MSI-positive cases, which reported a frequency of 100%, 97%, 98.7% and 100%, respectively. Altogether, these results suggest that *HSP110* is the most mutated MSI-target gene in MSI-positive CRC [14]. Importantly, in the discordant cases, all four MSI-H/IHC-positive cases presented alteration in *HSP110* (T17), and in the two MSS/IHC-negative cases and in the two MSI-L/negative-IHC cases the *HSP110* (T17) was also alterated, suggesting a higher sensitivity of *HSP110* (T17) to detec MSI compared to pentaplex, in agreement with recent findings of Buhard *et al* [32].

The *HSP110*, like other heat shock proteins (HSP), protects cells against adverse conditions, acts as a nucleotide exchange factor for HSP70 and acts as a major chaperone [45, 46]. Because of this activity, HSP110 is a good antigen-carrying protein and is used as an extracellular protein in the vaccine formulation [47]. Although, its tumorigenic properly is not fully understood, in CRC is suggested to preventing cells to entry in apoptosis [48].

Both techniques compared herein are routinely used and each one has advantages and disadvantages. IHC has the advantages of being applicable in formalin-fixed, paraffin-embedded tissues (FFPE), easy to perform, affordable, and able to guide the mutation screening of MMR genes. On the other hand, IHC can only assess a limited number of proteins, it is vulnerable to the quality of tissue preparation, and heavily depends on pathologist interpretation [39, 49]. The molecular technique has several advantages: i) it is applicable in formalin-fixed paraffin-embedded tissues (FFPE); ii) it does not need an expert pathologist for analysis; iii) it is objective and highly accurate compared to the subject evaluation of the staining pattern of the IHC slides; iv) it can identify MSI-positive tumors that present defects in MMR but have protein staining due to a non-truncated missense mutation or to mutations in other MMR proteins which are not included in the IHC panel; v) it is feasible in small biopsies [50]; vi) is less disposed to change than IHC following neoadjuvant and radiation therapy, and vii) it has a low cost per patient when the assay is performed in-house. However, it has the disadvantage of the need of a molecular genetic facility and specialized staff, and like the IHC, the FFPE pre-analytic issues, such as tissue fixation, can also interfere with PCR reaction [22, 37, 39]. In the present study we used an optimize in-house assay that does not need matched adjacent normal tissue [27], thus leading to a more cost-efficient assay when compared with four immunohistochemistry (MLH1, MSH2, MSH6 and PMS2) reactions. Despite of the similar results, our findings suggest that the molecular MSI assay, with the use of pentaplex plus *HSP110* (T17) marker leads to a more accurate MSI determination and it is less sensitive to pre-analytic issues in compared with IHC. Therefore, it is the ideal methodology for the routine assessment of MSI for CRC management in a low resource country as Brazil.

We observed an MSI-positive frequency of 10%, which is in line with international reports that vary between 12-16% of CRC patients [8, 51, 52]. The slightly lower frequency described can be due to several factors, such as: tumor staging; different criteria for inclusion of patients, different ethnicities of the patients’ analyzed, and environmental criteria that may affect the presence of MSI in the CRC.

In conclusion, the present study elucidated the MSI frequency in a robust manner by analyzing a large series of Brazilian CRC patients, who exhibited a known admixture ancestry [27]. Our results suggests that molecular analysis correlates better with the MSI phenotype in the CRC, and the *HSP110* (T17) improves the MSI determination in a routine setting of a Brazilian Cancer Hospital.

## MATERIALS AND METHODS

### Participants

In the present study 1013 CRC patients diagnosed between 2010 and 2014 at Barretos Cancer Hospital, Barretos, São Paulo, Brazil were enrolled. CRC patients were referred by the Departments of Digestive Surgery and Oncogenetic for MSI testing. Surgery-based cases presented CRC with no information of the hereditary context, whereas the Oncogenetic-based cases had confirmed or high clinical suspicion of familial CRC syndromes. Patients came mainly from the Southeast region of Brazil (77,7%), followed by Midwest (8,6%) and Northeast (8,1%), and few from the other Brazilian regions. Of the total of 1013 cases, 96.3% (976/1013) were Surgery-based cases and 3.7% (37/1013) were Oncogenetic-based cases, being 1.8% (18/1013) confirmed Lynch syndrome cases, 1.5% (15/1013) confirmed Familial Adenomatous Polyposis (FAP) syndrome cases and 0.4% (4/1013) of unclassified hereditary syndrome. Among patients, 52.1% were male and the mean age was 57.8 years (standard deviation 13.9 years). Concerning tumor location, 25.2% were right sided, 51.3% located at left colon/proximal rectum and 23.5% at middle rectum/distal rectum.

We also evaluated 214 healthy individuals from the BioBank at Barretos Cancer Hospital, Barretos, São Paulo, Brazil. The average age of the individuals was 33 years old, 52.3% were male, and 90% of the individuals came from the Southeast region of Brazil (São Paulo and Minas Gerais states), whereas others came from Paraná, Rio Grande do Sul, Bahia, Mato Grosso, Mato Grosso do Sul, Paraíba, Pernambuco, and Rondônia regions. This study was approved by the IRB at our institution (600/2012).

### DNA isolation

Tumor FFPE DNA were isolated using QIAamp DNA Micro Kit (Qiagen, Hilden, Germany) following the manufacturer’s instructions. DNA was isolated from 5-μm formalin fixed paraffin-embedded (FFPE) tissue slides. Briefly, tissues were deparaffinized by a serial extraction with xylene and ethanol (100%, 70%, 50%), and separately pathologist selected tumor areas were macrodissected using a sterile needle and carefully collected into a 1,5 mL PCR tube.

Blood DNA from healthy individuals was isolated using QIAamp DNA Blood Mini Kit (Qiagen, Hilden, Germany) following the manufacturer’s instructions.

### Mismatch repair immunohistochemistry

FFPE tissue blocks were cut into 3 μm sections for immunohistochemistry (IHC) using Dako EnVision™ FLEX detection system Kit (Dako, Glostrup, Denmark) and Autostainer Link 48 equipment (Dako, Glostrup, Denmark) following the manufacturer’s instructions. Antigen retrieval process was done at 97°C by 20 minutes (pH 9.0). Endogenous peroxidases were blocked with EnVision™ FLEX Peroxidase-Blocking Reagent (Dako, Glostrup, Denmark). The primary antibodies anti-human used in this study were: FLEX monoclonal mouse anti-MutL protein homolog 1 (MLH1) (clone ES05, ref IS079, ready-to-use, Dako, Glostrup, Denmark); FLEX monoclonal mouse anti-MutS protein homolog 2 (MSH2) (clone FE11, ref IR085, ready-to-use, Dako, Glostrup, Denmark); FLEX monoclonal rabbit anti-postmeiotic segregation increased 2 (PMS2) (clone EP51, ref IR087, ready-to-use, Dako, Glostrup, Denmark); and FLEX monoclonal rabbit anti-MutS protein homolog 6 (MSH6) (clone EP49, ref IR086, ready-to-use, Dako, Glostrup, Denmark). The DAB solution was used for immunostaining visualization. Slides were counterstained with hematoxylin. Nuclear staining of normal epithelial cells, lymphocytes, and stromal cells served as positive internal controls in each case. All cases were analyzed by one expert pathologist (CSN) who, based on nuclear staining, classified each protein by its expression. Regardless of the intensity or the extent of cell staining, the positive status was found for the cases that showed staining (presence of the expression of the protein under analysis) and the negative status when no staining was present (absence of expression of the protein under analysis).

### Molecular microsatellite instability (MSI)

The MSI evaluation was performed using a multiplex PCR comprising five quasi-monomorphic mononucleotide repeat markers (BAT25, BAT26, NR21, NR24, and NR27) as reported [27]. Primer sequences were described elsewhere [28]. Each antisense primer was end labeled with a fluorescent dye: FAM for BAT26 and NR21; VIC for BAT25 and NR27; and NED for NR24. PCR was performed using the Qiagen Multiplex PCR Kit (Qiagen, Hilden, Germany), with 1 μl of DNA at 50 ng/μl and the following thermocycling conditions: 15 min at 95° C; 40 cycles of 95° C for 30 s, 55° C for 90 s and 72° C for 30 s; and a final extension at 72° C for 40 min. PCR products were then submitted to capillary electrophoresis on an ABI 3500XL Genetic Analyzer (Applied Biosystems, Foster City, USA) according to the manufacturer’s instructions. The results were analyzed using GeneMapper v4.1 (Applied Biosystems, Foster City, USA) software to measure the fragment length in base pairs.

Cases with 2 or more markers out of quasi-monomorphic variation range (QMVR) were classified as MSI-H (MSI-High), cases with only one marker out of QMVR were classified as MSI-L (MSI-Low) and cases without markers out of QMVR were classified as MSS (Microsatellite stability), as reported [27].

### Determination of the *HSP110* (T17) QMVR

Blood DNA samples from 214 healthy individuals were used to determine the quasi-monomorphic variation range (QMVR) of *HSP110* (T17). The determined QMVR was tested in MSI-negative CRC cases (MSS=180 and MSI-L=37) and in MSI-positive CRC cases (MSI-H=90), with DNA samples extracted from FFPE cancer tissue.

PCR was performed using 5 μL of Qiagen Multiplex PCR Kit (Qiagen, Hilden, Germany), 1 μL of primers [1 mM] (Forward primer: 5’TGGGAAGTGTTCATGTGCTC3’ and Reverse primer: 5’TGAATCATGGTTCCAGATCAGA3’), 3 μL of water and 1 μl of DNA at 50 ng/μl and the following thermocycling conditions: 15 min at 95° C; 30 cycles of 94° C for 30 s, 55° C for 90 s and 72° C for 30 s; and a final extension at 72° C for 40 min. PCR products were then submitted to capillary electrophoresis on an ABI 3500XL Genetic Analyzer (Applied Biosystems, Foster City, USA) according to the manufacturer’s instructions and the results were analyzed using GeneMapper v4.1 (Applied Biosystems, Foster City, USA) software.

*HSP110* (T17) was also analyzed by R method, whose determination was calculated using the height ratios between peaks, namely R1 (between T14 and T16) and R2 (between T15 and T16), as described [31].

## SUPPLEMENTARY MATERIALS TABLE


