# Supplementary material for: Advantage of HSP110 (T17) marker inclusion for microsatellite instability (MSI) detection in colorectal cancer patients
Source: Oncotarget. 2018 Jun 19;9(47):28691–701. doi: 10.18632/oncotarget.25611 (PMC6033349; doi:10.18632/oncotarget.25611)
Supplement: Supplementary file 1 [file oncotarget-09-28691-s001.pdf]

Advantage of *HSP110* (T17) marker inclusion for microsatellite instability (MSI) detection in colorectal cancer patients

SUPPLEMENTARY MATERIALS

Supplementary Table 1: Summary of cases with discordant data between immunohistochemistry and molecular MSI analysis

Sample ID	Age	Primary Tumor Localization	Histologic Grade	Histologic Type	MMR Gene Mutated[#]	IHC status	Molecular MSI status	Molecular MSI alleles					*HSP110* (T17) status	*HSP110* (T17) alleles
								NR27	NR21	NR24	BAT25	BAT26		
11.0011	66	Colon	III	Adenocarcinoma	not performed	Negative	MSS	85/85	105/105	121/121	146/146	177/178	Altered*	131/131
12.0767	68	Rectum	II	Adenocarcinoma	not performed	Negative	MSS	85/85	101/105	122/122	145/145	177/177	Altered*	131/131
11.0226	46	Colon	II	Adenocarcinoma	*MHS6*	Negative	MSI-L	84/84	105/105	122/122	141/146	177/177	Altered*	132/132
13.0395	57	Cecum	II	Adenocarcinoma	not performed	Negative	MSI-L	83/83	103/105	122/122	141/147	177/178	Altered*	131/131
11.0404	55	Colon	III	Mucinous Adenocarcinoma	not performed	Positive	MSI-H	78/86	101/104	123/123	142/144	168/177	Altered	129/132
11.0827	64	Colon	II	Mucinous Adenocarcinoma	*MHS6*	Positive	MSI-H	82/84	98/104	120/123	139/141	167/174	Altered	129/132
13.0578	89	Colon	III	Medullary Adenocarcinoma	not performed	Positive	MSI-H	71/85	93/105	113/122	140/146	164/175	Altered	128/131
13.1023	46	Cecum	I	Adenocarcinoma	not performed	Positive	MSI-H	75/86	95/105	119/122	136/145	165/176	Altered	125/131

HSP110 (T17) status obtained by comparison between normal and tumor tissue DNA from the samples and also by R method.
MMR: mismatch repair; #: germline mutation; MSS: microsatellite stability; MSI-L: low-microsatellite instability; MSI-H: high-microsatellite instability; *: analyzed by R method.
